# Phenomenological psychology and qualitative research

**DOI:** 10.1007/s11097-021-09781-8

**Published:** 2021-10-30

**Authors:** Magnus Englander, James Morley

**Affiliations:** 1 Department of Social Work, Faculty of Health & Society, Malmö University, Malmö, Sweden; 2 Department of Psychology, Ramapo College of New Jersey, Mahwah, NJ USA

**Keywords:** Phenomenology, Psychology, Qualitative research

## Abstract

This article presents the tradition of phenomenologically founded psychological research that was originally initiated by Amedeo Giorgi. This data analysis method is inseparable from the broader project of establishing an autonomous phenomenologically based human scientific psychology. After recounting the history of the method from the 1960’s to the present, we explain the rationale for why we view data collection as a process that should be adaptable to the unique mode of appearance of each particular phenomenon being researched. The substance of the article is then devoted to a detailed outline of the method’s whole-part-whole procedure of data analysis. We then offer a sample analysis of a brief description of an ordinary daydream. This is an anxiety daydream in response to the recent Covid-19 pandemic. We present this daydream analysis in full to show the concrete hands-on 5 step process through which the researcher explicated the participants’ expressions from the particular to the general. From this brief sample analysis, the researcher offers a first-person reflection on the data analysis process to offer the reader an introduction to the diacritical nature of phenomenological psychological elucidation.

Pure phenomenology's tremendous significance for any concrete grounding of psychology is clear from the very beginning. If all consciousness is subject to essential laws in a manner similar to that in which spatial reality is subject to mathematical laws, then these essential laws will be of most fertile significance in investigating facts of the conscious life of human and brute animals.—Husserl 1917.^1^

The natural sciences were never intended to study man as a person. One need not leave the realm of science to study man adequately. We need only to broaden science itself.—Giorgi, 1970,2

## Introduction

Recently, there has been a healthy and long overdue discussion over how best to appraise the many new qualitative methods and how they contribute to scientific knowledge in psychology. For phenomenological psychologists the crucial challenge is, as expressed by Edmund Husserl (quoted above), to show how phenomenology provides a "*concrete grounding*" and "*fertile significance*" to the development of psychology as a science. Historically, it is well known that psychology, by and large, has imitated the methodology of the natural sciences. As expressed by Amedeo Giorgi (quoted above), by emulating physical science, psychology gave up studying human beings *"as persons*." In response to this critical flaw at the heart of modern psychology, phenomenological psychologists endeavor to redirect psychology toward a more phenomenologically based direction. The centerpiece of this project has been the development of a qualitative research methodology that would make a phenomenological psychological science possible. What follows is an outline of the original research method, where we also offer an example of data analysis as carried out by the researcher.

## Historical context: the project of a human science psychology

Before we launch into our main presentation, we believe that it is important to offer a brief historical review to illustrate the unique way in which this method developed in close collaboration with phenomenological philosophy. The following section is a synthesis that draws from historical accounts by Smith (2002), (2010), Cloonan (1995), and Churchill and Wertz’s (2015), as well as from the past experience of the authors.

In the early 1960’s Giorgi found phenomenology to be practiced in an ambivalent and often methodologically contradictory manner in European academic psychology. Similarly, American humanistic psychologists, sympathetic to phenomenology, were active critics of the deterministic approaches of mainstream psychology. But they, nonetheless, like their European counterparts, also defaulted to non-phenomenological measurement techniques when it came to their own research designs. It was as a response to this situation that the first systematically phenomenological psychology program was founded at Duquesne University in the early 1960’s. In this context Giorgi and his colleagues articulated this distinctly phenomenological way of doing psychological research—a methodology consistent with its phenomenological foundations. While Giorgi took the lead role in the development of this methodology, it needs to be stressed that this a was also an interdisciplinary community endeavor that took place between the philosophy and psychology departments at Duquesne University spanning the 1960’s to the late 1980’s. John Scanlon, the translator of Husserl’s *phenomenological psychology* lectures, was particularly supportive as a consultant to Giorgi and his colleagues during this period—as was Richard Rojcewicz, Al Lingis, Lester Embree, and several non-Duquesne but sympathetic scholars such as Martin Dillon, William Richardson and many others whom, records show, were often invited as guest speakers and consultants. Also, the psychology curriculum required students to take a minimum of two courses in modern philosophy, whereas the psychology faculty consistently audited philosophy courses.

In 1970 Giorgi launched the *Journal of Phenomenological Psychology*, which was at the outset a joint venture with European phenomenologically oriented psychologists and psychiatrists, as well as phenomenological philosophers. The journal was initially co-edited by Georges Thines and Carl F. Graumann. Serving on the first editorial board were Europeans such as Blankenburg, Buytendijk, Gurwitsch, van den Berg, van Breda, and Straus. The key point here is that the work being done on the development of the research methodology was part of a radically interdisciplinary and international project from the very beginning. As part of the overall project, Giorgi also founded the *Simon Silverman Phenomenology Center*. This research center also carries a copy of Husserl’s unpublished papers from the archives in Leuven, as well as the archives of Gurwitsch, Straus, Strasser, Bouman, Heidegger’s Marburg lectures, Buytendijk’s *Pensée Repensée*, and over 20,000 volumes, making it the largest collection of existential-phenomenological literature in the world. At the official inception of the center, Giorgi invited John Salis as his co-director.

Giorgi's seminal work, *Psychology as a Human Science: A Phenomenology-Based Approach* (1970) expressed a phenomenological response to the historical situation of psychology as a natural science. This also served as a foundational text for the psychology curriculum at Duquesne. Here, as a psychologist, he first proposed the necessity of a rigorously procedural, qualitative research method for a human scientific psychology. It made the appeal for an overall paradigmatic unity of “approach, method, and content” as the basis for a non-naturalistic psychology—an authentic *Geisteswissenschaft* or ‘human scientific’ psychology. Giorgi insisted that if psychology is to be true to its own subject matter, the scientific study of humans *as persons,* then the meaning of term 'empirical' in psychology must by necessity be 'broadened' beyond empiricism’s restriction to the sensory (see also, Giorgi, 1971, 2009). A phenomenologically empirical science would be inclusive of *all* experience. This would include (in Husserl’s terms) the *ir-real,* or the more than sensory aspects of experience, not just the *real* or sense-based measurables of classical empiricism. The vision was to employ the overall phenomenological paradigm to ground a human scientific psychology, a scientific enterprise autonomous from the naturalistic juggernaut of mainstream psychology.

Over this 50-year history this methodological approach has been known by various names: the phenomenological psychological method, the existential-phenomenological psychological method, the qualitative phenomenological method, human science psychology and even “the Duquesne method.” The founding Duquesne faculty mostly preferred the term “*Existential-Phenomenological Psychology*” to highlight the influence of *all* main continental thinkers: Heidegger, Sartre and Merleau-Ponty—as well as Husserl and many others. The term “existential” also expressed their emphasis on concrete psychological *situatedness* in contrast to transcendental phenomenological philosophy. Phenomenological psychologists who received their graduate training from within the Duquesne research tradition, such as, Frederick Wertz (Wertz et al., 2011) used the term “Phenomenological Psychological Method,” whereas Scott Churchill (2022) maintains the original Duquesne term “Existential Phenomenological Research.” As we will see ahead, it was only in 2009 that Giorgi committed to the nomenclature of “the descriptive phenomenological method in psychology.” The emphasis on description was done to offer a counterpoint to the penchant among qualitative researchers, often influenced by cultural postmodernism, to take the extreme position that 'everything is an interpretation'—something rejected by Giorgi as the imposition of a hermeneutic universalism (Giorgi, 1992).^3^ However, while generally based on Husserl’s approach, it is very important to highlight how in his 2009 text he never claimed his method to be identical to Husserl's. It was instead it was a *modification* of Husserlian philosophical methodology to adapt to the human scientific context of the discipline of psychology (Giorgi, 2014, 2021).^4^ In addition, Giorgi (2006, 2010, 2018) has also made several critical comparisons with other qualitative phenomenological methods as well as replies to philosophers (Giorgi, 2017, 2020, 2021). Several of his psychology colleagues and ex-students have developed variations of the method. Davidson (1988, 2003, 2021), for example, offers such a variation, to which both Giorgi (2020, 2021) and Wertz (2016) are sympathetic. Churchill (2022) maintains the core Husserlian elements while complimenting them with Heideggerian insights. But all such variations maintain most of the key components of the overall method—as shall be outlined ahead.

Across the development of this research tradition, there have been innumerable studies published in various psychology journals and books based on this overall approach. This research tradition is cited as a significant development within the history of modern psychology (see Brennan & Houde, 2017). Important theoretical and original qualitative research findings were published in the four volume, *Duquesne Studies in Phenomenological Psychology* (Giorgi et al., 1971, 1975, 1979, 1983), as well as the edited volume *Phenomenology and Psychological Research* (Giorgi, 1985). The latter contains paradigmatic empirical studies on learning (by Giorgi) criminal victimization (by Wertz), thinking while playing chess (by Aanstoos), and self-deception (by Fischer). A brief representative sampling that illustrates the range of recent research outputs is as follows: Living through positive experiences of psychotherapy (Giorgi & Gallegos, 2005), Lived persistent meaning of early emotional memories (Englander, 2007), Art appreciation (Roald, 2008), Pivotal moments in therapy (B. Giorgi, 2011), Postpartum depression (Røseth et al., 2011), Autism and culture (Desai et al., 2012), Leading a police vehicle pursuit (Broomé, 2013), Social anxiety (Beck, 2013), The suffering of older adults (Morrissey, 2015), The beginning of an extra-marital affair (Zapien, 2016), Mental health and the workplace (Tangvald-Pedersen and Bongaardt, 2017) Disturbances in maternal affection (Røseth and Bongaardt, 2019) Cross cultural learning (DeRobertis, 2017, 2020), and Black men’s experience of police harassment (Vogel, 2021).

## Data collection

Since this research tradition is oriented toward data analysis, this section on data collection will be brief and limited to some basic principles. Because psychologists are usually already well trained in interview techniques (Englander, 2020; Giorgi, 2020), it is natural that interviews will be commonly used to collect descriptive material. However, we stress that the method is not, by itself, an interview method.^5^ Instead, each data collection strategy is developed in an idiosyncratic way by first understanding how each phenomenon best reveals itself in its own unique mode of appearance (Englander, 2020). For instance, when studying ‘thinking while playing chess’ Aanstoos (1985), found interviewing, by itself, to be insufficient for accessing the subtle psychological nuances of playing chess. To accommodate this phenomenon, Aanstoos (1983), developed a 'think aloud method' where one player freely spoke his thoughts into a recorder during a chess game while the opponent had his ears covered. In other words, the principle here was to design the data collection process by attending closely to the particularity of the phenomenon. Typically, the phenomenon is carefully circumscribed in advance through pilot studies, field work and clinical contexts from which the researcher can uncover the ways to best solicit descriptions and expressions that can most successfully reveal deeper psychological meanings.

Our main point here is that there should be a ‘custom fit’ between the phenomenon and the data collection design to solicit maximally good descriptions of the phenomenon within the context of everyday life. Strategies for collecting such descriptions should not be presumed beforehand and imposed on the phenomenon. *The data collection design should fit the phenomenon instead of the phenomenon being forced to fit the design*. Concretely, the phenomenon or related phenomena should be carefully studied through the trial-and-error process of pilot studies before any final decisions are made regarding data collection strategies.

Having made these points, some general recommendations have been laid out for data collection procedures. Drawing from existential-phenomenological philosophers such as Sartre (1962, 28–29) and Merleau-Ponty (1962), phenomenological psychologists acknowledge that a person is always in a situation. At the start of any data collection, the research focus is on a *concrete situation* in which the participant has directly experienced the phenomenon under investigation. A concrete situation is not an idea, an attitude or anything abstract and conceptual—it is an experience that is directly lived. This acknowledgement of the situated *concrete* nature of psychological phenomena is another reason why data collection designs, again, need to be unique to the phenomenon and independently ‘custom-designed’ by the researcher. Or put another way, each study seeks the mode of investigation that allows the phenomenon to best express itself in its own distinctive way.

## Data analysis^6^

This is a ‘whole-part-whole’ qualitative method that includes steps where the researcher adopts the phenomenological psychological attitude and applies the technique of eidetic variation. Again, in contrast to philosophical analysis, phenomenological psychology begins and ends with meanings *as lived* and contextualized within the mundane, everyday lifeworld.

### Concrete 5 step method of data analysis

The data analysis has five steps. Over the course of nearly five decades of experience we have learned that success with this method is best achieved by applying each step in a generally sequential relation to the other steps. In this way, all five steps work as an integral whole. The steps that follow where adopted from a recent publication by Giorgi et al. (2017). Having said this, it is important to also point out that these steps have both a linier and *non-linier* dimension to them. The linear sequential ‘steps’ offers an initial structure and organization that can also liberate the researcher to move back and forth, reviewing previous steps and revising them in relation to new discoveries and intuitions. In actual concrete practice, the process becomes more like a working draft or scaffold to work from. Ahead, in our discussion of the case analysis, this non-linier dimension will be more fully addressed.

### Step 1. Initial reading for a sense of the whole

As this is a *whole-part-whole* method, the procedure begins with the ‘sense of the whole,’ proceeds with an analysis of the parts, and concludes with a newly elucidated ‘sense of the whole.’ Thus, the preliminary ‘appreciation’ of the entire description is important because it prepares and assists the researcher for the next steps where one studies its parts. This ‘sense of a whole’ should not be confused with hypothesis, conclusions or theorizations. Instead, it should be seen as a tentative understanding that is only an opening prelude to a *relationship* with the descriptive material. Importantly, it is this ‘sense of the whole,’ provided by the participant’s full descriptive account, that will act as the background to the diacritical figure-ground analysis carried out during the latter steps. In concrete practical terms, the researcher reviews the transcription (or audio or video) several times before starting Step 2. Again, this first step establishes the figure-ground framework that will drive the part-whole analysis of the entire method as every part, or meaning unit, will usually be explicated in terms of its relationship with the whole of the description.

### Step 2. Adopting the phenomenological psychological attitude

Adopting the overall phenomenological attitude or ‘way of seeing’ is what distinguishes this method from other forms of non-phenomenological qualitative research. Importantly, and this can’t be stressed enough from the onset, in our work as social scientists doing life-world qualitative research, the *epoché* and the reduction function in a different context then in philosophy.^7^ So, modified to accommodate the psychological sphere of interest, this attitude is essential to the next steps of the data analysis. Most would agree that time needs to be dedicated to the study authoritative primary sources in phenomenology to fully understand the nature of this phenomenological approach to research. This involves, (1) the *epoché* (or suspension) of the natural attitude, and (2) an assumption of the phenomenological psychological reduction.

With the practice of the *epoché* we try to just let the experience of something arise in its “givenness.”^8^ In Husserl’s terms this is a ‘putting out of play’ or ‘parenthesizing’ of any positions of belief or doubt toward the world as independent of our consciousness of the world. This ordinary everyday position towards reality is what phenomenologists call the ‘natural attitude.’ A corollary of the natural attitude is the *naturalistic* attitude which is the commonsense belief that all things are ultimately explained by the physical causes of natural science. So, the psychologist appropriates the *epoché* for several reasons, (1) it clears the way for us to better understand *how* the participants are experiencing the world, self and others, and (2) it liberates us to better describe other people’s experiences without falling back on physical explanations, rationalizations, stereotypes or explaining them away with hypothetical models and concepts. (3). It allows researchers to become more aware of how, as Merleau-Ponty (1962, p xiii) put it, one’s own ‘intentional threads’ are themselves influencing the phenomenon. (4). It invites researchers to overcome prejudices and doubts with regard to their own aptitudes for intuitive imagination. Put another way, the *epoché* opens us to see how the world is profusely intertwined with both the researchers and the research participant's experience of it, characterizing a radically non-dogmatic and open-minded perspective towards psychological research.

We will next go into some detail on the nature of the reduction in phenomenological psychology because it is here that phenomenological psychologists make significant and necessary modifications to the reduction, and in turn the *epoché*, as originally expressed by Husserl and philosophical phenomenologists. The phenomenological psychological reduction is what one does after first understanding the perspective of the *epoché.* Here we ‘reduce’ or restrict our frame of reference to a particular region of meaning. The psychological, in this sense, can be viewed as a particular region of science that is a *psychological* reduction. In the human scientific context of a qualitative psychology, a psychological reduction takes on a different meaning than Husserl’s original incomplete depiction of the psychological reduction. Husserl saw the psychological reduction as both a propaedeutic steppingstone towards the transcendental (or philosophical) reduction,^9^ as much as he also saw it as the basis for new kind of psychological science—as we are applying it here. However, not being a psychologist, Husserl was not able to offer detail on how to apply the psychological reduction in an applied human science context. It is here where Giorgi's modification of the psychological reduction incorporates the doings of science to qualitative psychological research. The psychological region pertains to a particular domain of lived experience—an experience that is neither abstractly conceptual, nor objectively physical; it is concretely and personally lived, by a particular person, always socially engaged, in a particular situation in everyday social life, in space, time and history.

In this sense, the psychological reduction maintains an intimate but distinctively delicate, even tricky, relationship with the natural attitude. While philosophers may be disinterested in the natural attitude in order to pursue other matters, the phenomenological psychologist is studying exactly the natural attitude itself. This mundane world of everyday common-sense beliefs is precisely the subject matter of the phenomenological psychologist—and any other phenomenologically identified social scientists. In this sense, the psychological position transforms the nature of the *epoché.* Instead of the philosopher’s full suspension of the world of the natural attitude, the psychologist takes strong interest in exactly this world of the natural attitude. This means that the psychologist performs an *epoché* that is both *in and out of* the natural attitude. Within the psychological reduction we ‘step back’ from the natural attitude in order to study its structures. Again, the phenomenological psychologist is cognizant of the faith of the assumed world of the natural attitude but still studies this worldview not unlike the empathic manner of an anthropologist, doing field work, who both spontaneously participates in village life, like a fellow villager, while also maintaining his social scientific perspective. So, unlike the faith of the participant, the researcher’s is a faith that regularly, and methodically, steps back and questions itself. These points will be further developed in our reflection on how this attitude, particular to the phenomenological psychologists, was applied to the data analysis process performed on our sample case description.

Another aspect of this circumscribed 'psychological' region is that it pertains to the domain of relevance that is, itself, the ‘discipline’ of psychology^10^ and what Giorgi (2009) has referred to as the 'disciplinary perspective'. Giorgi suggests that this ‘disciplinary’ reduction to the domain of the psychological (2009) should be most accurately depicted as a *human scientific* reduction.^11^ In stark contrast to the empirical theory of science that drives mainstream psychology, the approach provided here allows researchers to explicate psychological meanings in their morphological, provisional, phenomenological sense.

### Step 3. Dividing data into meaning units

This next step is motivated by practicality. Attempting to analyze, for example, 30–40 pages of transcribed interview material all at once is a daunting task. This is precisely why a data analysis *method* is helpful. Nevertheless, to stay consistent with a phenomenological theory of science, Step 3 is carried out from within the phenomenological attitude. For example, while reading through the recorded material, the researcher breaks down the material into smaller manageable parts to allow for a closer and more detailed focus in the upcoming Step 4. By phenomenologically elucidating the parts, the researcher is also able to begin distinguishing the participants’ meanings from how these appear in the natural attitude. This allows the expression by the participants to later (i.e., in Step 4) be explicated into phenomenologically psychologically sensitive description. The material is thus broken into manageable sections referred to as “meaning units.” The length of a meaning unit can vary from one sentence to an entire paragraph or (on rare occasions) a whole page of material. The length of meaning units can also vary from researcher to researcher, and such variation does not necessarily have any bearing on the general findings at the end of the analysis. Often the material can be easily differentiated. The main point is that too large a meaning unit can be unwieldy to analysis. It is also important to point out that not all meaning units are essential to the general structure of the phenomenon. However, all meaning units need to be analyzed (in Step 4). This last point is important, because sometimes when the researcher relaxes the *epoché* and returns to the natural attitude, some meaning units might mistakenly appear redundant. Nevertheless, when analyzed carefully, there is always the possibility of discovery.

Typically, researchers break this into two side-by-side columns that are written out in text form, referred to as *Column 1* and *Column 2*. This two-column transcription procedure serves several purposes. It conveniently organizes the process for the researcher and, importantly, it makes the data analysis process transparent and thus open for critique by other phenomenological researchers. As an additional procedure to this step, Giorgi also suggests that one modifies the participants’ expression into third person expressions. However, this is only a suggestion intended for researchers who are having difficulty in seeing the difference between the individual (or the idiographic level) and the phenomenon (the nomothetic level). Another discretionary modification is to extend columns, beyond the usual two, into three or even four columns. This was employed in the daydream analysis ahead where the researcher found a third column to be of value as it allowed him to visually check his more generalized transformations with the original meaning units—right before his eyes.

### Step 4. Transformation of everyday expression to psychological meaning

The relationship between *Column 1* (i.e., everyday expression, or naive description, of the participant) and *Column 2* (i.e., phenomenological description of psychological meaning) is distinctive to this method. Here one carefully elucidates the participants’ essential meanings into generalizable terms within the domain of psychological relevance—as expressed above. We grasp and draw out the fuller psychological meanings embedded within the everyday description. Now, it is in this particular step that the phenomenological attitude takes center stage and is explicitly put into practice for the purpose of a phenomenological psychological analysis. In addition, in order to seek the general meanings within the lived experience this step also includes the tool of *eidetic variation*. This means that the researcher needs to maintain a general focus on the phenomenon under investigation while carrying out this detailed analysis. In this context, phenomenological elucidation is not a matter of mere notetaking, summarizing, annotating or just condensing meanings. It is more about how the researcher adjusts one’s mindset so as to allow the psychologically relevant meanings to emerge to one’s consciousness. In a certain sense, one opens oneself, or renders oneself a vehicle to the fuller meanings of the participant’s naive description, but always with a focus on the phenomenon. This is a receptive or ‘discovery’ mode of consciousness—not one of actively applying ideas, theories or concepts. One can understand this position as a contemplative openness to the givens of the other’s experience as it emerges through the participants’ expressions. There is an imaginative participation in the subjects’ descriptions not unlike the engagement one experiences when reading a novel, a poem, or any act of expressive art. There is here an ironically 'focused openness' or put another way: a resolute receptiveness. One converts the participant’s expressions (as conveyed within the natural attitude) into phenomenologically clarified psychological meanings by carefully following the intentionality in the participants' expression. The watchwords here are: *elucidation, illumination, and explication.* Here, we do not *add* to what our participants say, instead we *bring forth* the fuller meanings.

In addition, one does not need to restrict oneself to only one column during the analysis. It is perfectly feasible for the researcher to extend the analysis of the initial meaning unit into several levels of elucidation—such as a column 3 or 4. As noted in the previous section on Step 3, this 4th step is also about the spirit of transparency in science (similar to how one shows one's work when doing mathematics). By extending the analysis into stages or levels of analysis, one is showing colleagues exactly how one has reached these extended levels of generalization.

### Step 5. Returning to the whole and moving toward the general structure

It is at this phase that the researcher moves from a part-whole eidetic analysis to a new focus on the whole again. But now we have a new whole, a whole that is the end result of this entire procedure. Remaining within the phenomenological psychological attitude, as described above, the researcher’s intimate engagement with the meaning unit analysis now becomes an act of synthesis of the parts together into what is usually a temporally sequential narrative. The watchword here is structure. A structure is understood in gestalt terms as a whole, but a whole composed only of essential parts. The idea here is that if one where to hypothetically remove one of the parts, then the rest of the structure would fall apart. Therefore, the researcher wants to be prudent to not overstuff a structure. A good structure should follow the elegance of simplicity—as much as reasonable. Furthermore, the features or constituent parts should be invariant. By invariant we do not mean universal or absolute. We are fully aware that human phenomena are contingent to history and culture. We only mean that an invariant psychological structure should “hold together” within this culture at this point in history. Within these parameters we think it reasonable that generalized psychological claims can be made.^12^

It is important to note that most other qualitative research methods present their conclusions in terms of ‘themes.’ But because this approach emphasizes phenomena as totalities, i.e. as structures, we avoid any overemphasis on themes and prefer to comment on the structure of the phenomenon as a totality as much as possible. When we do discuss parts, we prefer the term ‘constituents’ to stress their relatedness to the whole of the structure. It is conventional for many other methods to present to readers curated direct quotes from their participants. But because we have already performed a very close analysis of the direct expressions of the participants in the earlier steps of the data analysis, we prefer to offer readers the more structural, or general, levels of meaning in any discussion of our results as will be seen ahead when we discuss the results of our analysis of an experience of daydreaming. In short, our inclination is to offer readers prepared or explicated data instead of curated raw data.

### Situated structures

As an optional procedure one can add an extra step between the meaning unit analysis (step 4) and the General Structure (step 5). While Giorgi stressed the general structure, most advanced researchers find it effective to add this intermediary step—as demonstrated in the analysis offered ahead.^13^ This can support the eventual goal of generality and can be an extremely helpful ‘bridge step’ toward the general structural description. But it must be stressed that to remain only on the level of situated individual experience would miss the key purpose of the method—which is to achieve a general (inter-subjective) structural description of the phenomenon. Having said this, a situated structure can be very rich in life world details and remarkably illuminative in its own right. One could depict this as a structure on the idiographic or individual level. This is often popular with clinical psychologists who prefer an individual ‘case-study’ level of understanding. But unlike ‘clinical’ case-studies, this is a research phenomenon which is different from a diagnostic, or therapeutic relationship. Here the research intention is paramount—not the clinical intention. Again, this is the elucidation of an individual participant’s experience performed as a step before moving to the general structure. This would be *an essential structure of the invariant aspects of an individual person’s experience of the phenomenon.* In more simple language this is a basic summary of the psychologically relevant aspects of this particular person’s experience of the phenomenon. Developing situated structures from three or more research participants can be a very helpful way to eidetically scrutinize the phenomenon as experienced by all of the participants. But when it comes to groups, it is important to emphasize that within the phenomenological approach to science, eidetic comparison (Wertz, 2010) should not be confused with statistical comparison. Though more challenging (especially for newcomers), in phenomenological psychology an eidetic analysis could just as well be performed on a single participant as on a group. But having made this qualification, a group of any number of situated structures is always a great support to one’s eidetic analysis towards generalizability.^14^

### The general structure

**Fig. 1:**
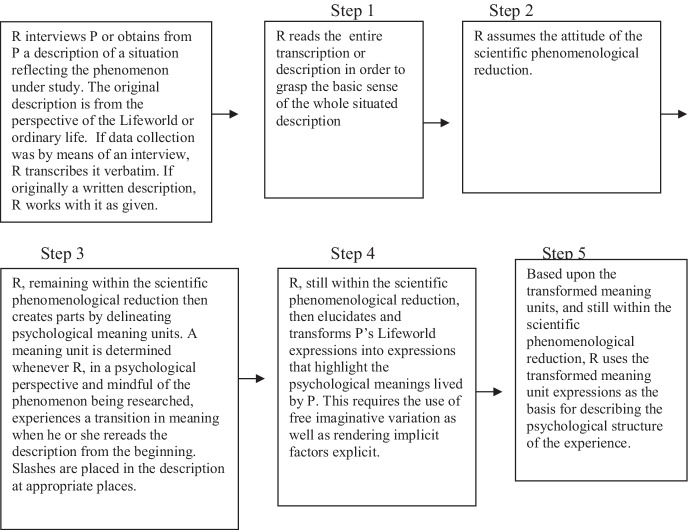
Overview—flowchart of data analysis process (from Giorgi et al., 2017). *R* researcher, *P* participant

At this point, these phenomenologically elucidated ‘parts’ of the data analysis (including the situated structures) are brought back together into a new *whole*. Phenomenological psychology is definitively a search for psychological essences or what we prefer to call general invariant structures. Husserl called this ‘eidetic analysis’ and the primary technique he used for this level of analysis he called eidetic or ‘imaginary variation.’ In this analysis, one imaginatively reviews the phenomenologically clarified parts of the previous analysis as achieved in step 4, with an eye for intuiting a new whole. Again, this is a discovery frame of mind where I render myself open to the continually emerging intuitions and patterns in the elucidated data as they give themselves to my awareness. In other words, it is not an empirical summary or the common denominator of facts across the cases, but another level of the analysis. Specific to this level of the analysis is the technique of imagining the phenomenon in its various profiles, angles or possibilities. For example, as a researcher I can ask myself if the structure of this phenomenon is possible without any of the particular constituent parts that I have discovered during my analysis in Step 4? I may even imagine adding new parts that were not explicitly expressed in the data but ‘apperceptively’ or intuitively suggested by the data. To reiterate, in contrast to most other qualitative approaches, the general structure is an integral whole and is never just a series of separate themes. The key idea here is that a structure is a full *gestalt*, a whole, or a totality that dissipates when a part is removed. Therefore, it is important to edit a general structure with rigor and integrity and to delete all that is unessential to the systemic pattern that makes the phenomenon what it is. The general structure is typically narrated in the present tense—though not always. Sometimes a phenomenon may split off into types or variants. In such cases one could have two or three general structures, representing different ‘types.’ Therefore, forcing a closure by applying a psychological theory is not an option. The findings, as supported by the analysis, can at a later stage in the discussion section (of the research report) be presented in dialogue with established psychological theories (‘backloading’ in current nomenclature) and other research results (See Fig. 1).

## Case example

What follows is a brief case example of a phenomenological psychological data analysis. Again, unlike philosophy where the research is done in a solitary first person manner, in phenomenological *psychology* we take a second person position. We see ourselves as *participants*—not mere observers—as we try to grasp the fuller meaning of other people’s concrete descriptions as expressed within the natural attitude of everyday life (Englander, 2020; Giorgi, 2009). We make no demands on our participants to take the reflective attitude of the practicing phenomenologist. Instead, only the researcher is responsible for taking the phenomenological stance as he or she reads the expressions of the participant. Here the data analysis is conducted within the tension of two intertwined goals: to be faithful to the *intentional* meanings as expressed while also deepening their meaning through their re-expression within the phenomenological psychological attitude—as performed in meaning unit analysis (step 4) and the development of structures (step 5). This, again, is what we call *elucidation* or *explication*. This is a fidelity that also takes us into a deeper understanding of the expressed intentions our participants. This is exactly the power of the *epoché* (within the psychological standpoint) as applied to the grasping or bringing-forth of psychological meaning. Like the way certain artists can transform the taken-for-granted experience of an ordinary object, such as an apple in a still-life painting, into an apple seen afresh ‘as if for the first time,’ so does the phenomenological psychologist strive to bring out the psychological meaning of the participant’s experience of the phenomenon.

The sample presented here is taken from the context of an ongoing research project on daydreaming that is currently replicating and updating a previously published study (Morley, 1998, 1999, 2003) through fresh interview material. As explained above, the data collection process was customized to suit the unique nature of the phenomenon. Here, in this particular research context, the procedure for collecting daydream reports has been to first request a self-written protocol from persons who are not themselves directly involved with psychology. A formal protocol question prompt (see below) was given to the participant to help guide the written description. As mentioned above, the reason for beginning with a written description is that, as an imaginary phenomenon, daydreaming can become unwieldy and difficult to articulate during an interview. Through pilot trials we have learned that written descriptions help the participant to ground or anchor their memory of the daydream. It then serves as an organizing point of reference for the interview—without imposing any leading external influences. Then, the researcher and participant begin the interview itself by re-reading the written protocol together to refresh their memories of the event. The researcher initiates the interview by asking the participant to take the initiative to express what, in the written description, he or she feels is most in need of elaboration or expansion. After the participants have offered further elaborations on what stands out as most important to them, the researcher will then pose questions from an informal semi-structured check-list of points of special phenomenological interest to the researcher. Specifically, the researcher asks for fuller descriptions of existential constants such as *space, time, embodiment, social relations, sense of reality,* and *sense of self* as experienced during the various temporal phases of the daydream. The actual interview approach, for this particular phenomenon, will vary across a spectrum from a gentle reiterative style to intensive and challenging inquiries^15^—depending on circumstances. As described above, this data collection method was developed through the researcher’s intimate relationship with the phenomenon over time.

A full data analysis of an entire interview would surfeit the space of this presentation. So it is for this reason that we chose to offer a concise sample of the analysis process drawn from material that was recently collected in the form of an initial written protocol. While not as detailed and spontaneous as the interview that followed, the written protocol still offers the reader a rich “sense of the whole’ that allows for a faithful sample the data analysis process. So, though brief, this was still a reasonably good description that offers a worthy example of the whole-part-whole dynamic central to the analysis process of this method. Choosing a brief sample also expresses the authors’ confidence that even the smallest fragment of an everyday type of description will explode in meaning when approached from within the phenomenological psychological attitude. Not unlike how the sensory empirical world burst open with the introduction of telescopes and microscopes, so does the human life world open up before us when beheld from within the openness provided by the lens of the overall phenomenological perspective as expressed above.

Having said this, we again caution that as a *sample* data analysis it does not benefit from the detail offered by the follow-up interview. This small sample is offered for strictly didactic reasons. More importantly, it also stands alone without the fuller dimensionality offered by the intersubjective eidetic analysis at least two other individual case examples to which it’s whole and constituent parts could be eidetically compared. It was for this reason that we restricted the title of the phenomenon from “daydreaming” to “an anxiety daydream” to reflect the particularity of the one sample. But even without the intersubjective corroboration of at least two other daydream descriptions, we hope readers will agree that it can be surprising to see what can emerge when using only one case example.

To reiterate, in brief, we begin with the whole daydream description as depicted in the written protocol. After reading for the whole we then break it into parts—or meaning units. Then, we phenomenologically elucidate each of the parts, or meaning units, though the technique of using columns—in this case we used 3 columns (most researchers only use two). Finally, we return to a renewed *sense of the whole* in both of the situated and general structures. The situated structure, like a case study, is idiographic to the particular description while the general structure is an attempt to achieve a nomothetic statement on the phenomenon of anxious daydreaming. In this instance, the general structure will be restricted to the meanings elicited from this single, and very brief, case example and will therefore be somewhat limited and tentative. It’s very important to note that in most research instances the general structure will be an eidetic analysis based on the various other individual situated structures. The general structure corresponds to what one could call the *results* of the research process. While the constituent parts of the whole structure will be discussed in most research reports, unlike most other qualitative methods that discuss *themes*, typically supported with selected quotes, we prefer to keep the *whole* structure of the experience as the primary reference point.

Ahead, within the analysis we will refer to the participant as ‘P.’ Later, in the discussion, we will address the participant through the pseudonym of Ashling.

### Written daydream protocol—initial protocol prompt to the participant (P)

Please concretely describe a situation in which you experienced a daydream. Please describe what was happening when the daydream began, what the daydream was about, what it was like while having the daydream, and how the daydream came to an end. Please try to be as concrete and detailed in your description as possible.

### Ashling’s written protocol description—including step 3, marking the meaning units

On March 14, 2020, I was in Tepoztlán, Mexico. Trump had recently announced he would be suspending travel from Europe to the US due to COVID-19. I had just moved to Mexico a few months prior. I feared if the closure was happening with Europe it would most likely be happening with Mexico very soon, a golden moment for Trump to assert his plan for the border with Mexico to be even more impenetrable. As we drove back from Tepoztlán to Mexico City and night was falling, I started to gaze out the window, daydreaming, as we passed the silhouetted Popocatépetl volcano in the distance.

I started thinking about how I would get back to my family in the US if flights were suspended with Mexico. As we continued to drive I thought about if we didn’t stop in Mexico city but just continued all the way to the border (about a 15 h drive). In my daydream I imagined arriving at the border and that there would be mayhem, cars piled up for miles and the border patrol not allowing anyone across. The border agents were armed and aggressive and unreachable. I imagined the reasons I would give, that my family needed me etc., but reasoning with them was not working. And I envisioned somehow managing to get past them as they were distracted by the chaos, and the relief felt by speeding into the US away from the border and onward towards home.

I felt anxious imagining the border patrol and their dominance, their potential to shoot us when we sped past, defying their rules of closure. But I then felt relief at the outcome of getting past, of fighting our way in and across and making it to a place of safety.

When my partner and I later got to the apartment in Mexico City that night I looked into flights to get to Boston where we would be in a familiar place during this most intensive and uncertain time. My good friend called me from Rennes in France and told me how bad it was, that death rates were rising, and how she wasn't leaving the house at all. She advised me to leave quickly and that to have a garden was a saving grace for her, and that at least in Boston I would have a garden. I booked my flight and packed a small case. I daydreamed again as I looked around the apartment, that 10 or so years would pass, and I would finally be able to come back and all my things would be here but between and around old weeds and crumbled walls and cobwebs, a scene left untouched and abandoned.

### Meaning unit analysis

**Table:** 

Colum 1 Exact language of the participant expressed in the 3rd person. (3rd person is optional)	Colum 2 The researchers’ psychological elucidation of the participants expressions	Colum 3 The researcher’s further psychological elucidations. (extra columns are optional)
Meaning Unit (MU) 1 On March 14th 2020, P was in Tepoztlán, Mexico. Trump had recently announced he would be suspending travel from Europe to the US due to COVID-19. P had just moved to Mexico a few months prior. P feared if the closure was happening with Europe it would most likely be happening with Mexico very soon, a golden moment for Trump to assert his plan for the border with Mexico to be even more impenetrable.	P is an American who has recently moved to Mexico City. P has been aware of how the borders are closing in Europe due to the imposition of quarantine conditions. She has been *fearful* that this virus will come to Mexico very soon. Furthermore, she is also aware of how particular aspects of American political forces could make this border closing especially ominous and *impenetrable.*	The spreading corona virus border closings are making P feel the threat of losing her access to her home in another country. She is feeling constricted by these forces beyond her control.
MU 2 As P and her partner drove back from Tepoztlán to Mexico City and night was falling, P started to gaze out the window, daydreaming, as they passed the silhouetted Popocatépetl volcano in the distance.	At the moment, P is driving with her partner, in a car, returning from a weekend holiday in a country village outside of Mexico City. Night is falling and there is a dramatic landscape in the horizon drawing her attention away from the interior of the car. Gazing out the window P’s attention goes towards the horizon of the twilight landscape.	The immediate situation does not allow her to express her strong feelings of fear and anxiety. She focuses her attention to the external distant horizon. Her attention shifts to a new field.
MU 3 P started thinking about how P would get back to her family in the US if flights were suspended with Mexico. As they continued to drive, she thought about if they didn’t stop in Mexico City but just continued all the way to the border (about a 15 h drive).	The daydream is initiated by P’s concerns about the practical problem of how to get back to her family in the USA if flights are suspended. As they continue on with their driving, P thinks about not stopping in Mexico City and, instead, continuing the 15 h drive all the way to the USA border.	The daydream is initiated in a certain sequence. From refocusing her attention to the distant horizon away from the car interior, to her practical concern over the problem of whether or not she can book flights, to now being on the imaginary international border.
MU 4 In P’s daydream P imagined arriving at the border and that there would be mayhem, cars piled up for miles and the border patrol not allowing anyone across.	P’s attention shifts from the landscape to another scenario—that is imaginary. Here she imagines driving past their actual destination. Instead she imagines having driven all the way to the international border. Entering the daydream, P finds herself as already arriving at the border. There is a scenario of *mayhem*. Cars are piled up for miles.	An imaginary scenario appears in a way that manifests her fears of entrapment. P is the active agent at the creative source of this world scenario but is also in the role as the suffering victim of these circumstances of chaos and entrapment.
MU 5 The border agents were armed and aggressive and unreachable.	This scenario of great disorder is created by the authorities who forbid access to the border crossing. These repressive authorities are dangerous, incommunicative and unresponsive to any reasoning.	
MU 6 P imagined the reasons P would give, that her family needed her etc., but reasoning with them was not working.	P tries to persuade the border agents to let her cross, but they do not respond and remain unreachable.	The dominating and dangerous authorities are unresponsive and offer no opportunity for negotiation or satisfaction. She is trapped in a situation of complete impasse.
MU 7 And P envisioned somehow managing to get past them as they were distracted by the chaos, and the relief felt by speeding into the US away from the border and onward towards home.	She next *envisions* circumventing the impersonal and threatening border agents. Taking advantage of their distraction due to the chaos, she speeds the car past them, away across the border towards home. *Getting past* the border guards *gives relief.*	Within this daydreamed world scenario, P now shifts from impassive victim of circumstances beyond her control to taking the initiative to *get past* the authorities and drive across the border without waiting for their official permission. This decision gives P a feeling of *relief*. She is on her way home. Doing what she wants to do. Going where she wants to go. She has made a transition from powerless to empowered.
MU 8 P felt anxious imagining the border patrol and their dominance, their potential to shoot us when we sped past, defying their rules of closure.	Imagining the border patrol illuminated her own feelings of *anxiety*. They exuded dominance and the threat of harm as they circumvented the guards and sped past them.	As daydreamed, P is fully aware of the risk she is taking and the potentially dangerous consequences of this defiance of their rule and authority.
MU 9 But P then felt relief at the outcome of getting past, of fighting our way in and across and making it to a place of safety.	The outcome of fighting their way across and getting past the border guards gives a feeling of relief. They enter *a place of safety.*	The daydream concludes with an overall feeling of relief and the satisfying sense of safety that P has been longing for.
MU 10 When P and her partner later got to the apartment in Mexico City that night P looked into flights to get to Boston where they would be in a familiar place during this most intensive and uncertain time.	In the aftermath of the daydream experience, P takes action by making inquiries into securing a flight to her home (in Boston) which will be a familiar place to be during this time of uncertainty.	The daydream opened P to the fact that she wanted to go home, and she takes concrete action to make this happen.
MU 11 P’s good friend called her from Rennes in France and told P how bad it was, that death rates were rising, and how she wasn't leaving the house at all. She advised P to leave quickly and that to have a garden was a saving grace for her, and that at least in Boston P would have a garden.	A friend in France contributes to her anxiety by telling P about the increasing pandemic rates in Europe and how the house quarantine makes having a garden very important. She is advised to leave quickly for home in Boston where she will have the comfort of a Garden instead of being restricted to the confines of an apartment where she is in Mexico.	A social interaction reinforces her desire to fly home to Boston.
MU 12 P booked her flight and packed a small case.	P now fully follows through on her decision to depart Mexico by booking her flight and packing her case.	Further supported by the social interaction, she makes her decision final.
MU 13 P daydreamed again as she looked around the apartment, that 10 or so years would pass, and P would finally be able to come back and all her things would be here but between and around old weeds and crumbled walls and cobwebs, a scene left untouched and abandoned.	P has another daydream of returning to her Mexico apartment after 10 years of being away.	

### Situated structure of an anxiety daydream

Daydreaming for this person was an imaginary manifestation of her feelings of anxiety. By manifesting this anxiety as a dramatically staged scenario, she was able to live-out or play-out the enactment of her anxiety and its eventual resolution. This particular daydream occurred as a person’s affective response to the threat of having her freedom of movement, across international borders, curtailed or restricted by political forces beyond her control. In particular she feared being cut-off and separated from her home and family during a time of great uncertainty. These strongly felt emotions around the experience of constraint or restriction had no means of expression within the context of a long road trip in a car. Turning her gaze, away from the car interior, out the window towards the twilight horizon of the landscape, P entered into an imagined scenario where she is in the same car but has arrived at the international border between her foreign country of residence and her desired home country. The daydream manifests the person’s own momentary existential situation as a scene of chaos and mayhem enforced by the imposing, threatening and impersonal agents of power i.e. the border guards who refuse to allow her to cross the border into her home country. P imagines trying to reason or negotiate with the guards but realizes that dialogue is futile in this situation. Again, these are circumstances out of her control. As a staged enactment or ‘metaphorization’ of her actual existential situation, the daydream is both the expression and revelation of her life situation. It allows her to “express” her immersion in the situation which also, in a reversible way, offers her a reflective distance to “see” the feeling of restriction that has occupied her. As both the expression and revelation of her present life situation the daydream is, in this sense, lived ambiguously as both an active and passive experience. These ambiguously dual, yet interwoven, perspectives are implicit to her daydreaming experience. Next, within the imaginary narrative of the daydream, the daydreaming/daydreamed person commits an act of defiant transgression. P shifts the narrative from that of passive casualty of powers beyond her control, to one where she takes charge, or assumes agency, by choosing the extreme risk of speeding past the distracted guards and thus flouting their overbearing authority by driving across the border without their sanctioned permission. By taking matters into her own hands and transgressing the rules, P escapes confinement and experiences the satisfaction that comes with the security of having returned to her home country. The daydream concludes with feelings of relief. The experience of this daydream allowed P to articulate her desire to return home to her native country during this time of uncertainty—a desire that was converted into an actual concrete decision to eventually book an airline flight home to family and friends.

### Tentative general structure of an anxiety daydream

Daydreaming emerges in a situation of unfulfilling circumstances. In the case of anxiety, it appears in the form on an ominous and yet opaque threat to one’s well-being. This feeling presents itself as a demand for action—to seek the source of the threat and to overcome it. However, this demand for action cannot be achieved in the current situation as it is impeded by circumstances where no real behavioral action is possible. This becomes a tension between the feeling’s demand for action, regarding the ominous threat, and its restraining context. The person turns attention away from the immediately restraining situation by seeking out and shifting attention to another horizonal field of focus. It is here that the emotion takes the course of expressing itself through the medium of an imaginary scenario that opens up an opportunity for the fulfillment of the emotion. The emotion transforms into a world scenario where it is expressed in the form of an enacted narrative drama. The person assumes a dual intentional role as both the author/narrator of the dramatic scenario and well as the actor immersed within the dramatic action. The emotion is now lived in a narrative context that allows the possibility of its fulfilment. As a staged enactment the daydream can become a living metaphor of the person’s actual existential situation. The daydream scenario can be both the *expression* and *revelation* of one’s emotional situation. Its expression makes it possible to “see” one’s immersion in the emotional dramatic scenario. It can offer the opportunity for a reflective distance from the feeling of restriction that had previously occupied the person. As both expression and revelation of the person’s present life situation daydreaming reveals an ambiguous interplay between both active and passive aspects of experience. These ambiguously interwoven perspectives vary between being implicit or explicit to the daydreamer. Though daydreaming takes place within an imaginary region of experience, this region is always also interfused within one’s life historical horizons—always expressing one’s life projects and goals.

## Commentary on the analysis

In any phenomenological psychological research report, there is an extensive theoretical discussion of the results (i.e. the constituent parts of general and situated structures) with the phenomenological and natural scientific literature. We have *much* to say here, especially with regard to such constituents as ‘dual intentionality’ ‘multiple realities, the ‘affective-imaginary dynamic,’ the “linkage of expression with revelation’ and, of course, the comparison of these findings with current studies in cognitive science (such as the default mode network). But alas, as the purpose of this essay is didactic with regard to the method, and due to the limits of space, we must defer this full dialogue to a future publication.

Due to the brevity of the written description, and the very fact of there being only a single participant, the researcher can only modestly offer a highly tentative sample general structure. However, despite its brevity, the participant, whom we will here call ‘Ashling,’ offered a rich and full description and the researcher feels confident that the situated structure was faithful to the participants experience.

### The non-linier dimension of data analysis

While the researcher initially worked with fidelity to the 5 step method, it is also important to note that there was a significantly non-linier dimension to this process. This was especially the case when it came to the composition of the situated and general structures. Once the meaning units were demarcated, the process towards the situated and eventual general structures took on a life of its own. In other words, while the meaning units established a framework for data analysis, once the 3 column framework was established, and the participant’s expressions were laid out *before his eyes*, the researcher began a back-and-forth process of checking, rechecking, reflecting and intuitively linking the meanings into fuller wholes and patterns. To use an imperfect metaphor, we can compare this explication process to what is called a detective’s “crazy wall” that is used to help interpret and understand a crime case. From detective stories and movies, we are familiar with how the investigator will post pieces of data and information across a wallboard, or sometimes a city map. The detective can then use this to meaningfully link the information and datapoints with connecting strings. Seeing the constituent parts ‘before his eyes’ helps the investigator to make the ‘meaningfully intuitive connections’ that lead to better understanding of the case. Obviously, this helps the investigator to step back and see the dynamic relation between the parts and the whole and it is from this perspective that insights and discoveries can arise. This is exactly the benefit of meaning unit analysis.

### The diacritical aspect of data analysis

To reiterate, the psychological phenomenological attitude is focused on understanding the particular experience of a particular person. Obviously, as evidenced by the general structure, we do not stop a the particular—but this is where we begin. While this attitude undoubtedly suspends the naturalistic attitude of physical science, its disposition towards the more global natural attitude, as discussed above, contains a strategic ambiguity. Very importantly, unlike phenomenological philosophy, phenomenological psychology directly takes up the naively believed world of the natural attitude as a subject of inquiry. Ours is, as Maurice Natanson, citing Alfred Schutz, calls it: “a phenomenology of the natural attitude” (1973, p107). In other words, while we ourselves as researchers are trained to be aware of our own natural attitude, and ‘step back’ from it as best we can, it is also true that we do not entirely put it aside. So, for example, when reading Ashling’s description of her daydream, the researcher imaginatively participated with the description of her daydream and, for that moment, may have been empathically engrossed within the world of her natural attitude. In a recent publication this is well described by Scott Churchill as a ‘disciplined fascination’ (Churchill, 2022). Also, as a denizen of the natural attitude oneself, the researcher may well have applied his background stock of knowledge of daydreaming, garnered from personal experiences as well as professional readings on the subject; all of this in order to better understand Ashling’s experience and intentional structures. Hence, as discussed above, this is not a *pure epoché* or a *pure* reduction as practiced by the philosopher. On the other hand, unlike Ashling, or any research participant, the researcher continually practices a ‘stepping back’ from that believed world, again, in order to better understand her world. There is, in this way, a *weaving process* that is unique to the phenomenological psychological attitude.

The figure-ground metaphors used by Merleau-Ponty are very helpful here. Throughout his works he explicitly describes what we are calling the phenomenological psychological attitude, as a ‘*diacritical*’ process (Kearney, 2011) that is, like the act of breathing—both inhaling and exhaling as one whole act. This is precisely what we mean by the strategic ambiguity of the phenomenological psychological position. In his well-known discussion on methodology Merleau-Ponty describes the attitude of the researcher as follows: “Reflection does not withdraw us from the world…’ “…it steps back to watch the forms of transcendence fly up like sparks from a fire; it slackens the intentional threads which attach us to the world and thus brings them to our notice…” (1962, p xiii). As psychologists these threads or tethers to the natural attitude are never cut, they are “loosened or slackened” to enable us to see the intentions of others—as well as one’s own. Seeing my own intentional threads can reveal fore-understandings that could either *inhibit* or *enhance* my analysis.

In this case, a young woman is learning about the encroaching covid pandemic, wants to return to the security of her home and family, and becomes upset about the closing international borders that could restrict, and become an obstacle, to her desire to return home. This was the big picture to which the researcher returned, in a circular manner, throughout the analysis.

The researcher came to see how Ashling was originally overcome with a desire to go home while simultaneously experiencing a feeling of being impeded from that intention. Though she did not explicitly say this, one could easily imagine how, as more borders closed, Ashling’s desire to return home would only intensify. The beginning part of the daydream narrative reflected this distressing and overwhelming devils circle where she is impeded by powers beyond her control. But in meaning unit 7 we see a turn.

Another diacritical element is the weaving between the whole of the description and its parts. As a reader one could say that I am “zooming-in” on the unique and minute details of the participants expressions as much as I am continually “zooming-out” to use the whole as the context for understanding these details. For example, Ashling’s use of key expressions in Meaning Unit 7 (MU7) such as “envisioned,” “getting past” and “the relief felt” all offered a basis for enhanced eidetic exploration and fuller illumination. They allowed the researcher to come to the insight of Ashling’s shift in position, from that of passive victim of overpowering circumstances to that of an active agent of an imaginary act of courageous transgression—driving past the armed and aggressive border guards to cross the border. Understanding the “whole” of her situation is what brought to light the essential meaning of the daydream.

### Spelling out tacit meanings

By *explication,* or *elucidation,* we mean the process of spelling-out latent or tacit meanings. To offer an example, Ashling, of course, never explicitly said that she experienced a ‘dual intentional structure.’ It was the task of the researcher to cull out this structural component that was implicit to the description and likely lived-out in a pre-thematic way by Ashling. The researcher’s recognition of this constituent happened during the researcher’s transition from the meaning unit analysis to the whole of the situated structure. It was in this process of “putting the whole story back together again” that the researcher saw how this double intentionality was experienced by Ashling. Here, there were two distinct but related intentions, (1). the intention to deal with the practical frustrations of booking a flight home during an uncertain period of international crisis (the actual world), and (2). the daydreamed intention of getting past imaginary border guards (the daydreamed world scenario). The researcher came to see Ashling as experiencing both intentions and both corresponding world relations—the *actual* car scenario and the other being the *daydreamed* car scenario. Hence, the dual intentional structure. One could call this a “generalizing process” but, in actual practice, it was a much fuzzier and more unclear event than any such nominalizations can portray. Once again, we can understand this as a diacritical process: (1). The insight came ‘as given’ in the discovery manner of a direct phenomenological intuition, and (2). This pattern was ‘recognized’ from the researcher’s background stock of knowledge (or fore-understanding) as a daydream researcher and reader of phenomenological literature. Because this elucidation process is itself somewhat pre-reflective, one can never have absolute certainty over whether it was an intuitive given or a pre-understanding.

Again, Merleau-Ponty’s diacritical approach helps to illuminate this elucidation process. In describing Merleau-Ponty’s (1968) diacritical approach to grasping meaning, Kearney cites James Joyce’s statement that it is possible to have “two thinks at a time.” (2011, p 1). Directly addressing psychological research, Merleau-Ponty says: “One may say indeed that psychological knowledge is reflection but that it is at the same time an experience. According to the phenomenologist (Husserl) it is a material *apriori*. Psychological reflection is a “constatation” (a finding). Its task is to discover the meaning of behavior through an effective contact with my own behavior and that of others. Phenomenological psychology is therefore a search for the essence, or meaning, but not apart from the facts.” (Merleau-Ponty, 1964, p.95).

With the term “constatation’ Merleau-Ponty is suggesting that both *observing*, (receiving the intuitive givens) and *asserting* (actively applying one’s stock of knowledge) can be at play in the same act of psychological understanding. Both are one whole movement within the same act—in the chiasmatic, reversable manner of a figure-ground dynamic. While space does not allow us to develop this issue in the detail it deserves, we raise this matter to try to bring some light to the act of elucidation that is so central to this method. The take home point here is that, while the method highlights the significance of description, this does not mean that one needs to choose between stark antinomies such as description and interpretation, or phenomenology and hermeneutics as within this elucidation process of ‘disciplined fascination’ both movements come together.

## Conclusion

### Towards dual disciplinary citizenship

This method was designed to give psychological researchers an organized and structured framework for doing second person research. The whole-part-whole process, in itself, is not complicated or difficult to understand and learn. What is difficult for those who are beginning this style of research, is the assumption of the phenomenological psychological attitude. This attitude, which distinguishes this method from non-phenomenological qualitative research methods, can’t be taken for granted and requires training, study, and the support of a like-minded research community. Because it is founded in phenomenological epistemology, phenomenological psychology is a hybrid discipline. The practice of phenomenological psychology requires a kind of ‘dual citizenship’ in both psychology and phenomenological philosophy. Those trained solely in philosophy’s orthodox emphasis on textual exegesis may often lack experience in practical professional life-world applications as well as an overall knowledge of the literature and scientific history psychology. On the other hand, those trained solely in psychology, with little to no exposure to philosophy, coupled with the field’s strictly naturalist experimental orientation—which underscores the natural/naturalistic attitude—come to phenomenology with this resilient attitudinal disadvantage that can take effort to overcome. What we have here, in the current academic world, is a set-up for mutual misunderstanding between these disciplines. While the sharp disciplinary divides of the current academic world make such ‘dual citizenship’ training difficult and rare, this is possible, but only with special effort and unique pedagogical interventions. There are institutionalized training programs, usually schools of psychotherapy, that are open to such interdisciplinary training. Yet, these programs are few and far-ranging in their offerings. Most independent researchers entering this field need to supplement their training in naturalistic psychology with an intense period of philosophical study of primary sources and guidance in this study is too often lacking. Then, on the other hand, it is encouraging to see the increasing number of philosophers who are taking an interest in “applied phenomenology.” Yet, we currently see little cognizance, in much of this recent literature, of the 50-year phenomenological psychological research tradition. We mention this, as a friendly invitation to psychologically interested philosophical researchers to acquaint themselves with their predecessors to avoid re-inventing the wheel and duplicating research results and techniques that have already been developed within the phenomenological psychological research tradition. In the same breath, we would just as strongly urge our colleagues in the social sciences to give more serious study to the phenomenological philosophical tradition.

## Footnotes

1 From Husserl’s inaugural lecture in Freiburg given 1917 and published in *Husserl—Shorter Works* (1981, 17).

2 This quote is from a talk that Giorgi gave at the *Symposium on science and scientism: the human sciences* Trinity College, May 15–16, 1970 and documented by Maurice Friedman (1984) *Contemporary Psychology: revealing and obscuring the human*. Pittsburgh: Duquesne University Press. (p.30).

3 To Giorgi, relativism is as much a dogmatism to be avoided in psychology as is reductionism. Giorgi's (2009) method, hence, became known as the descriptive phenomenological psychological research method. With the emphasis on description Giorgi intended to apply the phenomenological attitude by staying true to discoveries from the everyday lifeworld. So even though discoveries may sometimes be incomplete, he preferred that they were described in their incompleteness rather than forced into unnecessary closure for aesthetic or ideological reasons (ibid.). Hence, both psychologically relevant aspects of Husserl's phenomenology as well as the discovery-oriented spirit of science became essential influences on Giorgi's approach to the project of a qualitative research method in psychology.

4 Initially influenced by Merleau-Ponty’s psychologically oriented thought, Giorgi turned more to Husserl’s methodological emphasis in his pursuit of a phenomenological theory of science to support a qualitative psychological research method (see Giorgi, 2009). As Giorgi (2014, 236) recently stated "…I use Husserl because he confronts the issue directly and he contrasts his position with that of the empiricists." In the late 90’s, several other qualitative methods using a phenomenological approach started to emerge, most had a stronger emphasis on postmodernism or hermeneutics. Giorgi differentiated his method from the newer ones by stressing that his was a more descriptive emphasis as opposed to an interpretative one (Giorgi, 1992, see also, Giorgi 2006, 2010, 2018). Of course, the distinction should not be understood too literally, because in certain settings the use of the word ‘interpretation’ could synonymously refer to the act of ‘description.’ However, with the term ‘description’ Giorgi (1992) simply meant to *stay true, or rooted, to what appeared in the data*. This is similar to what is called a “close reading of the text” in literary studies. The intention was to avoid the kind of intrusive and overly imposing 'interpretations' where gaps in the qualitative data would be 'filled' with theoretical explanations, abstractions or even speculations.

5 Developing phenomenological interviewing skills requires practice and training that is often already present in the education of most clinical psychologists and health care workers. However, phenomenological psychologists have been recently applying the insights of philosophical phenomenology to better articulate the role of empathic reflection in participant observation (Englander, 2020; Churchill 2010) and designing phenomenologically inspired teaching methods (Englander 2014; Churchill, 2018) for improving quality of psychological interviewing and qualitative phenomenological research generally.

6 For a chronological development of the methodology, see Giorgi (1975a, 1975b, 1985, 1997, 2009, 2018).

7 Referring to Schutz, Michal Barber points out how these terms are “analogous to the phenomenological prototype.” In other words, again, as social scientists we apply them with a different purpose than that of the philosopher. See: Barber, Michael, "Alfred Schutz", *The Stanford Encyclopedia of Philosophy,* Summer 2021 Edition, Edward N. Zalta (ed.), URL =  < https://plato.stanford.edu/archives/sum2021/entries/schutz/ > .

8 The history of phenomenology could be considered one big ongoing deliberation about the meaning and possibility of the *epoché.* We hope readers will forgive us for sidestepping these discussions for the purposes of this presentation where space only permits us to present the *epoché* as practically applied to the research process in phenomenological psychology. But we will make this one brief point. All major phenomenological themes such as embodiment, temporality, intersubjectivity and even the hermeneutic circle were developed by philosophers thorough their initial employment of the *epoché*—or awareness of the natural attitude. It is therefore important, we believe, for one to understand the practice of the *epoché* to, in turn, fully grasp these phenomenological concepts. We find it inconceivable that one could proficiently comprehend basic phenomenological concepts such as the lived body or intersubjectivity while remaining unreflectively within the influence of the natural attitude. Similarly, we have learned through experience that success with the method we are presenting here is often in direct proportion to one’s awareness of their natural attitude.

9 The relation between the transcendental and the psychological reduction is another long-deliberated issue in the history of phenomenology which we can’t develop here. In brief, because the transcendental “philosophical” reduction is a non-personal and non-situated level of reflection it is simply not appropriate for performing qualitative psychological research—at the *moment* that we are doing it. To our knowledge, no phenomenological psychologist would claim to be doing both standpoints at once. But this does not mean that psychologists must, or should, ignore the insights of transcendentally derived philosophical concepts when we design our research or reflect on the results of our psychological analysis. Phenomenological philosophy can be a perfectly compatible basis from which to deepen our understandings of the results of our descriptive analysis. In short, psychologists may visit the transcendental position, but we do not unpack our bags, and we always remember our return ticket.

10 This is very similar to the relevance structure of a world as suggested by Schutz (1962).

11 As Giorgi (2009, 99–100) writes, “The researcher does, of course, assume the human scientific (psychological) reduction. Everything in the raw data is taken to be how the objects were experienced by the describer, and no claim is made that the events described really happened as they were described. The personal past experiences of the researcher and all his or her past knowledge about the phenomenon are also bracketed. This bracketing results in a fresh approach to the raw data and the refusal to posit the existential claim allows the noetic-noematic relation to come to the fore so that the substratum of the psychologist's reality can be focused upon. That is, the particular way in which the describer's personal acts of consciousness were enacted to allow the phenomenal intentional objects to appear from the basis of the sense determination that the psychologist is interested in uncovering.”.

12 For a more elaborate discussion on general knowledge claims in qualitative research and its relation to a phenomenological theory of science, see for example, Englander (2019).

13 Giorgi originally included situated structures but later dropped them to emphasize the nomothetic (or generalized knowledge) aspect of the method. But most Giorgi’s colleagues and ex-students prefer to include situated structures as a transition to the general. As teachers we have learned that this psychologically rich transitional step is of great pedagogical value. For most newcomers to the method, it is intuitively much easier to construct situated structures before moving on to develop general structures. We also find situated structures to be of great psychological value in their own right—as we hope is demonstrated in our case example ahead.

14 It is important to note that *research participants* are not considered from the stance of an empirical theory of science. Any qualitative methodology, grounded in a phenomenological theory of science, cannot naively adopt the concept of *the population* (and *sampling methods*) as its ground for making general knowledge claims (see for example, Englander, 2019).

15 At points in the interview when a more active questioning is called for, *evocation* techniques like those from the explication interview, or the micro phenomenological interview method, can be very effective. (see Petitmengin et al., 2018) Here, we invoke the daydream so that both the interviewer and the participant can, in an almost trance-like way, imaginatively re-live the daydream together. These techniques can provoke profoundly rich description. Here is another example of how we approach data collection as always contingent to the manner in which the phenomenon best expresses itself. Again, this is why we endorse an *adaptable* approach to data collection.
